# Exploring the In Vivo Fate of β-1, 3/1, 6-Glucan Using Quantitative Tandem Mass Spectrometry Based on a Structure-Specific Fragment

**DOI:** 10.3390/md23040177

**Published:** 2025-04-20

**Authors:** Shuying Xu, Jiale Hao, Chunyan Ye, Xintong Li, Pengcheng Gao, Ni Song, Chanjuan Liu, Youjing Lv, Guangli Yu, Guoyun Li

**Affiliations:** 1 Key Laboratory of Marine Drugs (Ministry of Education), Shandong Key Laboratory of Glycoscience and Glycotherapeutics, School of Medicine and Pharmacy, Ocean University of China, Qingdao 266003, China; 17syxu@alumni.stu.edu.cn (S.X.); hjl9700@163.com (J.H.); ye18716113504@163.com (C.Y.); lixintong20000723@163.com (X.L.); gaopengchengsir@163.com (P.G.); nisong1975@ouc.edu.cn (N.S.); liuchanjuan@ouc.edu.cn (C.L.); lvyoujing1988@163.com (Y.L.); 2 Laboratory for Marine Drugs and Bioproducts, Qingdao Marine Science and Technology Center, Qingdao 266237, China

**Keywords:** β-1, 3/1, 6-glucan, oligosaccharide-marker, UPLC-MS/MS, method validation, in vivo fate

## Abstract

β-glucan, a promising drug candidate for immuno-antitumor therapy, holds tremendous potential for clinical applications. However, the absence of highly sensitive quantitative methods for polysaccharides, attributed to their complicated chemical structures and susceptibility to endogenous interference, has posed significant challenges for their clinical development. Here, we report a highly sensitive and reliable analytical strategy for quantifying β-1, 3/1, 6-glucan derived from *Durvillaea antarctica* (BG136) in various biological matrices. This approach integrates targeted depolymerization and derivatization, followed by oligosaccharide isomer fingerprinting using ultra-high-performance liquid chromatography-triple quadrupole tandem mass spectrometry (UHPLC-MS/MS). The absolute quantification of BG136 relied on the abundance of the structure-specific trisaccharide (Glc-β1, 6-Glc-β1, 3-Glc) generated. This methodology not only facilitates prototype-based BG136 administration but also exhibits remarkable sensitivity. Following method optimization and validation, we successfully explored the in vivo fate of BG136 across multiple models, including cellular uptake and release kinetics, as well as preclinical and clinical pharmacokinetics. These achievements provide insight into the “black box” of BG136 from administration to elimination in vivo. This work marks the first practical application of this strategy in complex biological matrices, offering methodological support for the successful execution of the BG136 Phase I clinical trial.

## 1. Introduction

β-glucan is an important innate immune activator that is widely distributed in fungal, bacterial, algal, and plant cell walls. Emerging data in recent years suggest that β-glucan combined or conjugated with immune checkpoint inhibitors can simultaneously enhance innate and acquired immunity, leading to a more effective anticancer treatment [1,2,3]. The potential of β-glucan in pharmaceuticals has spurred extensive research into its pharmacokinetics. For example, lentinan, with an average molecular weight of 432.6 kDa, predominantly accumulates in the liver for metabolism and clearance [4], whereas yeast-derived particulate β-glucan preferentially targets the pancreas [1]. Pharmacokinetic studies of β-glucans predominantly focus on those with higher molecular weights due to their superior biological activity and drug-carrying capacity. In contrast, the pharmacokinetic profiles of lower molecular weight β-glucans have received less attention. BG136 is a β-1, 3/1, 6-glucan derived from *Durvillaea antarctica*, with a molecular weight range of 3–6 kDa. In 2022, it was approved for clinical trials (clinical approval no.: 2022LP02021) and is expected to be the first immune-antitumor marine polysaccharide drug in China. Previously, our team developed a UHPLC-MS/MS-SIR method for quantifying intact BG136, achieving an LLOQ of 0.5 μg/mL. However, this method lacks the sensitivity required to detect trace levels of BG136 in human biofluids at clinically relevant doses. Thus, there is an urgent need to develop more sensitive analytical approaches for accurate measurement of BG136 in clinical samples.

Developing sensitive and reliable analytical methods remains a major technological challenge in advancing polysaccharide-based therapeutics, primarily due to their complex chemical structures, heterogeneity, and molecular weight dispersion [5,6]. Current approaches for studying the cellular kinetics and pharmacokinetics of polysaccharides primarily utilize visual tracing techniques, such as fluorescent labeling [7,8,9,10]. However, this strategy alters the structure of polysaccharides via covalently linking small-molecule ligands prior to administration, potentially affecting the accuracy of in vivo behavior assessments. Although fluorescent or isotope-labeled polysaccharides can be employed for pharmacokinetic assessments in animal studies, their application in clinically healthy human subjects is significantly constrained. Therefore, developing a robust quantitative method based on native polysaccharides is crucial. This approach ensures that the in vivo transport process from administration to elimination remains unaffected by external factors, allowing all observations to be unequivocally attributed to the polysaccharide-drug itself.

The primary quantitative methods currently available for the prototypical administration of polysaccharide include chromatography and bioassays. Due to their lack of UV-absorbing groups, polysaccharides can only be analyzed using size exclusion chromatography coupled to an Evaporative Light-Scattering Detector (ELSD) or Refractive Index Detector (RID) [11], a method without sufficient detective sensitivity and consequently deemed unsuitable for clinical pharmacokinetics. In contrast, bioassays are recognized for their high specificity and sensitivity. For example, the “G-test” specifically recognizes β-1, 3-glucan with exceptional sensitivity, making it a gold standard for the clinical diagnosis of invasive fungal infections. Based on a similar principle, Yoneda et al. developed a sandwich enzyme-linked immunosorbent assay to quantify lentinan levels in plasma samples, achieving a sensitivity of 17 pg/mL [12]. However, previous studies from our laboratory have revealed that the G factor possesses a strong affinity for high-molecular-weight β-1, 3-glucans while showing no binding to BG136. Furthermore, a significant challenge in bioassays is obtaining an effector that strongly reacts or binds to the substance being measured. Therefore, applications of this approach in polysaccharide pharmacokinetics are limited.

Milestone advancements in mass spectrometry have revolutionized drug quantification, especially through multiple reaction monitoring (MRM) that assigns parent-daughter ion pairs as a drug’s unique identifier [13,14]. Although LC-MS techniques fall short in directly detecting macromolecular polymers, a promising avenue involves developing a quantitative method using a bottom-up approach [5,15]. This strategy has been widely commercialized in proteomic analysis for an extended period [16]. While there is growing interest in applying this strategy in the field of glycan analysis, its current advancement is largely constrained to quantitative analysis in aqueous solutions or food matrices [17]. To date, there have been no successful applications of this strategy in complex biological matrices, such as body fluids or tissues. The discrepancy in matrix environments significantly impacts the difficulty involved in quantitative polysaccharide analysis. This is because the interference from complex biological matrices is substantial and even a decisive factor for establishing a polysaccharide quantitative method [18]. Endogenous sugar chains, coupled with exogenous carbohydrates and dietary fiber ingested by organisms, contribute to a variety of neutral oligosaccharides and their isomers in biological organs and body fluids. Especially when the bottom-up strategy is applied to neutral polysaccharides like β-glucan, the resulting specific fragments encounter numerous endogenous and exogenous oligosaccharide isomers, yet the effective separation of oligosaccharide isomers is undoubtedly a formidable challenge [17,19,20,21]. Furthermore, certain unidentified components in complex biological matrices may inhibit enzyme activity, directly impeding the generation of oligosaccharides, which is a necessary first step in bottom-up analysis. Therefore, a singular protocol may not uniformly analyze polysaccharides across diverse biological samples, necessitating customization for different biological matrices.

We hypothesize that: (1) BG136 contains at least one structurally unique fragment that can serve as a quantitative marker; (2) biological matrices have varying interferences that affect the production and detection of these markers, making a single protocol inapplicable to all matrices; and (3) BG136 may trigger unique biological processes and in vivo fates involving biodistribution, biotransformation, and bioclearance compared to reported higher molecular weight β-glucans. In this study, the absolute quantification of BG136 relied on the abundance of the structure-specific trisaccharide (Glc-β1, 6-Glc-β1, 3-Glc). In plasma, the main interferences for BG136 analysis are trisaccharide isomers and high glucose levels, while urine contains additional inhibitors of enzyme activity. To tackle these issues, we developed two sample preparation protocols for accurately determining BG136 levels in various biological matrices. This methodology shows excellent sensitivity, enabling the precise quantification of BG136 in cellular, animal, and human studies.

## 2. Results and Discussion

### 2.1. Optimization of Sample Preparation

A prerequisite for applying the bottom-up strategy to detect polysaccharide-drug in complex biological samples is ensuring that it remains intact in vivo or is degraded only in trace amounts, thereby maintaining the accuracy of the results. To investigate the feasibility of using this strategy to track the in vivo behavior of BG136, we initially assessed its form in urine after a single intravenous injection. The finding, displayed in Figure S1, suggests that BG136 is mainly eliminated in its original, unchanged form.

**Figure 1:**
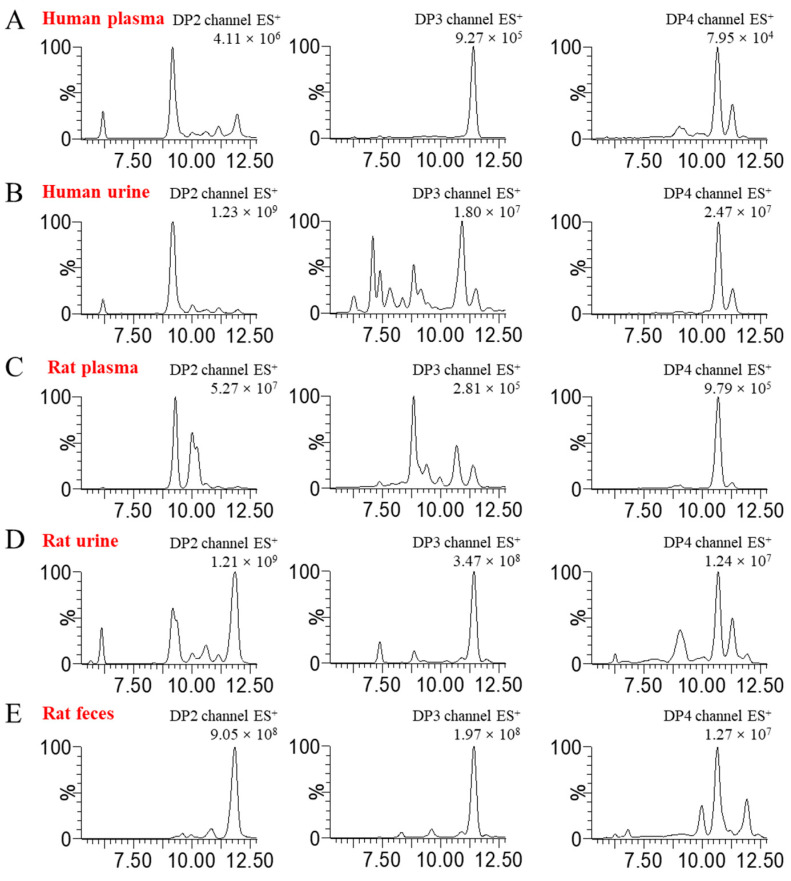
Oligosaccharide compositions in various blank biological matrices. (**A**,**B**) DP2–4 oligosaccharide profiles of human plasma and urine; (**C**–**E**) DP2–4 oligosaccharide profiles of rat plasma, urine, and feces.

Given the importance of dissociating polysaccharides into oligosaccharides for quantitative analysis, we utilized endo-1, 3-β-D-glucanase to completely degrade BG136. The enzymatic hydrolysis predominantly yielded disaccharides, trisaccharides, and tetrasaccharides (Figure S2A), designated as BG-Dis, BG-Tris, and BG-Tetras. Through targeted enzymatic hydrolysis and subsequent purification processes, we prepared standards of these fractions, achieving 99.08% purity for BG-Tris (Figure S2B). To improve ionization efficiency, various labeling reagents, including 2-aminobenzoic acid (2-AA), 1-phenyl-3-methyl-5-pyrazolinone (PMP), and AMAC, were tested during method development. Isomer interference, which arises from MRM overlap due to common parent-daughter ion pairs and similar chromatographic elution, presents a significant analytical challenge [22]. The hydrophobic nature of AMAC altered the polarity of the oligosaccharides, facilitating the separation of BG136-specific oligosaccharides from endogenous isomers in biological samples [23]. The parent-daughter ion pairs for the oligosaccharide standards are detailed in Table S1. The oligosaccharide profiles in disparate blank biological matrices are displayed in Figure 1. It is evident that the degree of isomeric interference of oligosaccharides varies across different biological matrices, illustrating a complex and multifaceted scenario.

**Figure 2:**
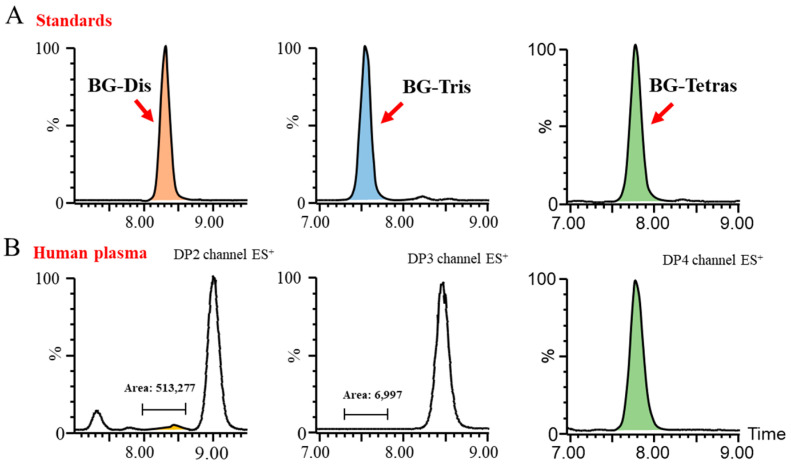
Evaluation of the specificity of BG136 hydrolyzed fragments for human plasma. (**A**) Typical chromatograms of BG-Dis, BG-Tris, and BG-Tetras; (**B**) DP2–4 oligosaccharide interference in BG136-free human plasma.

Subsequently, we endeavored to achieve the optimal baseline separation of BG-Dis, BG-Tris, and BG-Tetras from their corresponding isomers in blank plasma by refining the liquid phase conditions. A comparison of the AMAC-labeled oligosaccharide profiles of BG136 revealed a unique trisaccharide exhibiting high specificity. As presented in Figure 2, the presence of highly responsive isomers in blank plasma complicates the high-sensitivity quantification of BG-Dis and BG-Tetras. In contrast, BG-Tris not only achieved complete separation from the trisaccharide isomers but also exhibited relatively minimal interference at its retention time. Furthermore, BG-Tris constitutes the largest proportion in the BG136 glycan chain, accounting for 35.48 ± 0.97% (Table S2). Notably, BG-Tris distinctly stands out in terms of its specificity and abundance within the glycan chain of BG136, making it a reliable marker for accurate quantification. As depicted in Figure S3 and Table S3, nuclear magnetic resonance characterization has confirmed its structure as Glc-β1, 6-Glc-β1, 3-Glc (Figure S2C).

Moreover, we observed that the interfering substances present in various biological matrices are not identical. Hence, it may be essential to implement different pretreatment operations for different biological samples when aiming to detect BG136 content in distinct biological matrices. For instance, in blood samples such as plasma, serum, and high-sugar cell culture media, the high concentration of glucose can hinder labeling efficiency by competing for derivative groups. Consequently, the use of glucose oxidase becomes necessary to modify glucose and prevent interference during AMAC labeling. However, this consideration is not needed for tissues, urine, feces, and cells, where interference from glucose is minimal and negligible. Furthermore, when using the validated method for quantifying BG136 levels in plasma to analyze those in urine and feces, we were astonished by the absence of BG-Tris production. This anomaly was attributed to unidentified components in urine and feces that inhibited endo-1, 3-β-D-glucanase activity, which were evidenced to be small-molecule acetone-insoluble components. The intricate trisaccharide fingerprints present in blank urine and feces lead to an elevated chromatographic baseline unsuitable for highly sensitive quantitative detection. To solve these problems, we incorporated an ultrafiltration step, which not only effectively removed the enzyme-interfering components but also significantly reduced the oligosaccharide interferences in the biological matrix. As a result, we successfully established a reliable quantitative method for measuring BG136 levels in urine and feces.

Given the abundance of neutral carbohydrates in food, their digestion and absorption during meals can result in a notable increase in neutral oligosaccharide levels in the bloodstream [24]. Therefore, we examined the impact of subjects’ dietary status at the time of plasma collection on the quantitative analysis of BG136. The findings, as shown in Figure S4, demonstrated that fasting or feeding conditions during sample collection influenced the abundance of pre-existing isomers in plasma without introducing additional interference with BG-Tris. This observation once again underscores the structural specificity of BG-Tris. Furthermore, the addition of a salt solution during deproteinization proves beneficial for the quantitative analysis of BG136.

### 2.2. Optimization of LC-MS/MS Analysis Conditions

During method development, we systematically evaluated various chromatographic columns, column temperatures, mobile phase compositions (including salt types and concentrations, pH, etc.), and eluent gradients. Ultimately, we employed a C18 column for reversed-phase chromatographic separation of the analytes, fine-tuning the elution gradient to maximize the separation of BG-Tris from the isomers present in the blank matrix or introduced during sample pre-treatment. For instance, recognizing that urine samples contain a greater number of trisaccharide isomers than plasma, further optimization was performed based on the elution gradient of the plasma sample. Moreover, the mass spectrometry settings were fine-tuned to enhance the ionization efficiency of the analytes.

### 2.3. Method Validation

#### 2.3.1. Selectivity, Linearity, and Calibration Curve

**Figure 3:**
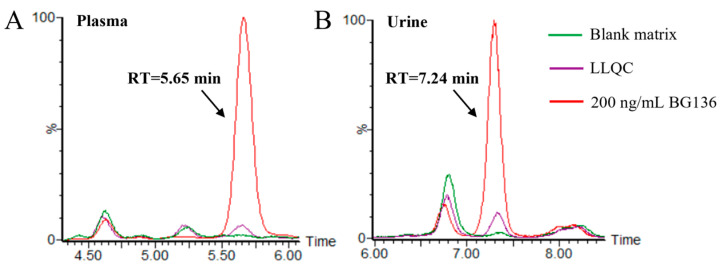
Selectivity evaluation of the method. (**A**) Typical chromatogram of drug-free human plasma and plasma spiked with 10 ng/mL BG136; (**B**) typical chromatogram of drug-free human urine and urine spiked with 20 ng/mL BG136. The green line and purple line represent the blank matrix and corresponding LLOQ samples, respectively.

Typical chromatograms of blank matrices and the corresponding lower limit of quantitation (LLOQ) samples are presented in Figure 3. No residues were observed following the analysis of high-concentration BG136 samples. The calibration curves for BG136 were assessed over the ranges of 10–250 ng/mL in human plasma, 20–1000 ng/mL in human urine, and 2.5–50.0 μg/g in rat feces, using least-squares regression analysis. The typical calibration curve equations for BG136 in various matrices are exhibited in Table S4.

Current mass spectrometry-based approaches for direct polysaccharide analysis in plasma show limited sensitivity. For example, the LLOQ for the quantitative analysis of dextran in rat plasma using total ion fragmentation coupled with Q-Orbitrap-MS technology was 3 μg/mL [25], while that for the authentication of β-glucan via integrating magnetic molecularly imprinted polymers and UHPLC-qTOF-MS analysis was reported as 10 μg/mL [26]. Although pharmacokinetic studies in animal models may overcome this challenge through higher administered doses, the clinical setting requires more sensitive detection methods due to lower drug levels. Although bottom-up strategies have gained traction in glycan analysis, their current applications remain restricted to aqueous solutions and food matrices. For instance, Lentinan has a reported quantification limit of 61.5 ng/mL [27], while that for polysaccharide from Danggui Buxue Tang was 67.7 ng/mL [28]. In this study, the LLOQs of BG136 in plasma, urine, and feces were 10 ng/mL, 20 ng/mL, and 2.5 μg/g, respectively. This disparity highlights two key advantages of our method: (1) the pioneering application of a bottom-up strategy to complex biological matrices and (2) the improved sensitivity of the strategy to quantitatively analyze glycan chains.

#### 2.3.2. Precision and Accuracy

Within- and between-run accuracy and precision at LLOQ, LQC, MQC, and HQC concentrations in various matrices are summarized in Table 1. The data showed that the recoveries of LLOQ and QC samples tested satisfied standard requirements, with mean values within 90.1% to 112.3% of the nominal values. For each concentration level, the intra- and inter-batch RSDs were less than 12.11% and 9.70%, respectively. These findings indicated that the accuracy and precision of the analytical method meet the acceptance criteria for biological samples.

#### 2.3.3. Matrix Effect

The evaluation of matrix interference in drug-free human plasma and urine involved a comparison of the peak areas of the post-spiked standards with those of the neat standards at LQC and HQC concentrations. As shown in Table 2, the RSDs of matrix factor normalized by IS in human plasma were 7.85% at LQC and 5.47% at HQC, respectively. Similarly, those RSD values in urine were 9.80% and 3.11% at 50 ng/mL and 750 ng/mL, respectively.

#### 2.3.4. Stability

The stability of BG136 in human plasma and urine was evaluated. As delineated in Table 3, the stability results under various storage and handling conditions met the acceptance criteria outlined in the guidelines for bioanalytical method validation. Analyte-spiked human plasma and urine samples underwent three to five freeze-thaw cycles. The recovery rates for plasma samples spiked with BG136 at 25 ng/mL and 225 ng/mL ranged from 100.6% to 102.5%, with RSD values below 2.52%. For urine samples spiked with BG136 at 50 ng/mL and 750 ng/mL, recoveries varied from 92.5% to 101.7%, with RSDs less than 5.16%. Overall, BG136 remained stable after at least three freeze-thaw cycles in both human plasma and urine.

Subsequently, BG136-added plasma and urine samples were refrigerated at 4 °C for 20 days and 7 days, respectively. The recovery rates of plasma samples spiked with BG136 at LQC and HQC ranged from 101.2% to 109.7%, with RSD levels below 4.64%. Similarly, the recoveries of urine samples spiked with BG136 at LQC and HQC were 108.8% and 96.8%, with RSD values of 2.96% and 3.49%. These results indicate that BG136 can be stored at 4 °C for at least one week.

Ensuring the stability of post-preparative samples in the assay environment is critical for result accuracy. Hence, processed plasma and urine samples were placed at ambient temperature for an extended period. After 14 days at 22.5 ± 2.5 °C, the post-preparative high- and low-concentration plasma samples exhibited recoveries ranging from 95.6% to 102.7%, with RSDs below 7.45%. Similarly, the recoveries of BG136 in post-preparative urine samples were within 98.7% to 102.7%, with RSD values being inferior to 7.47%. These findings indicate that the post-preparative samples have a long shelf life at room temperature for over a week.

### 2.4. Cellular Uptake and Release Kinetics of BG136

**Figure 4:**
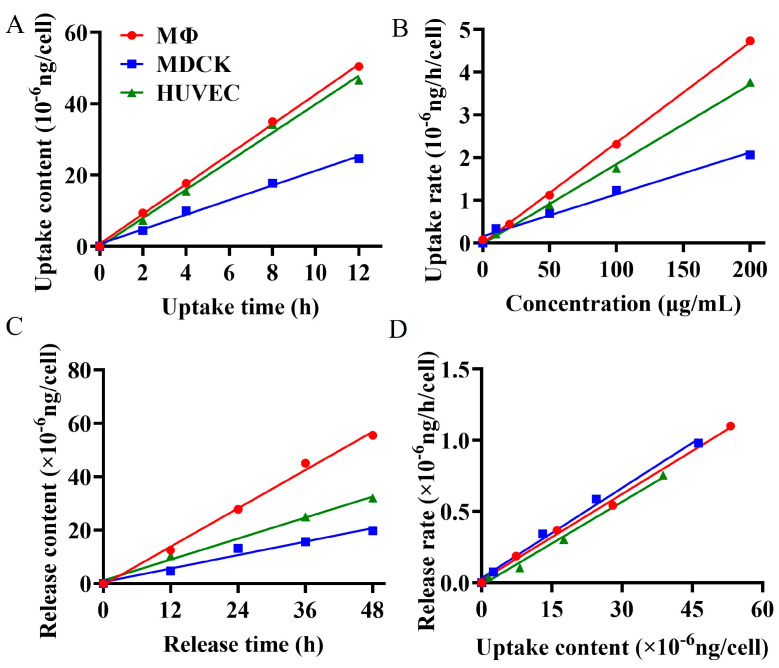
Cellular uptake and release kinetics upon exposure to BG136. (**A**) The curves of the uptake contents for individual MDCK, HUVEC, or RAW264.7 macrophage cells over incubation times; (**B**) the curves of uptake rates with varying incubation concentrations; (**C**) the curves of effluent contents from single cells over incubation times; (**D**) the curves of release rates based on uptake contents.

The cellular internalization process directly influences the bioavailability and therapeutic efficacy of drugs [29,30]. Researchers currently can only qualitatively judge the cellular internalization of polysaccharide drugs through visual tracing [1,31,32], leaving a gap in quantitatively revealing the cellular uptake or release kinetics. A comprehensive understanding of the kinetics of cellular uptake and release of polysaccharide is essential for designing precise drug delivery systems [33]. The uptake and release processes from epithelial and endothelial cells reflect the drug transport in vivo, while the interaction between immune cells and β-glucan plays a pivotal role in their immunomodulatory function [34]. To quantitatively elucidate the phagocytosis and exocytosis patterns of BG136 in epithelial cells, endothelial cells, and immune cells, we utilized the established quantitative method to measure changes in intracellular drug content at various concentrations, exposure durations, and release times. The findings, as depicted in Figure 4, demonstrate that these three types of cells exhibit time- and dose-dependent uptake and release of BG136. The internalization and externalization processes followed a first-order kinetic model. The net uptake constants were measured at 2.36 × 10^−11^ mL/h in RAW264.7 macrophages, 1.87 × 10^−11^ mL/h in HUVEC, and 9.80 × 10^−12^ mL/h in MDCK. Correspondingly, the release constants were determined to be 0.0211 h^−1^, 0.0195 h^−1^, and 0.0202 h^−1^, respectively. Based on this kinetic model, internalized and externalized β-glucan levels can be accurately assessed to achieve desired effects by adjusting parameters such as drug concentration and exposure time.

Cell size significantly influences drug uptake capacity [35]. Generally, larger cells possess a greater cell membrane surface area, which enhances their ability to uptake drug molecules more efficiently. This factor likely contributes to the higher uptake of BG136 by HUVEC cells compared to MDCK cells. Macrophages, known for expressing multiple pattern recognition receptors on their surface—such as C-type lectin receptors (CLRs) and Toll-like receptors (TLRs)—can specifically bind to β-glucan [34,36]. Previous studies have shown that TLR4 binds to BG136 and mediates its entry into macrophage cytosol [37]. Despite their smaller size, RAW264.7 macrophages have the highest uptake rate of BG136, likely due to pattern recognition receptors on their surface. This endocytic pathway is both efficient and exclusive to immune cells. The release constants of BG136 from RAW264.7 macrophages, MDCK, and HUVEC cells were nearly identical, suggesting that the release pathway or mechanism of BG136 is likely shared among all three cell types.

### 2.5. Pharmacokinetic Properties of BG136 in SD Rats

**Figure 5:**
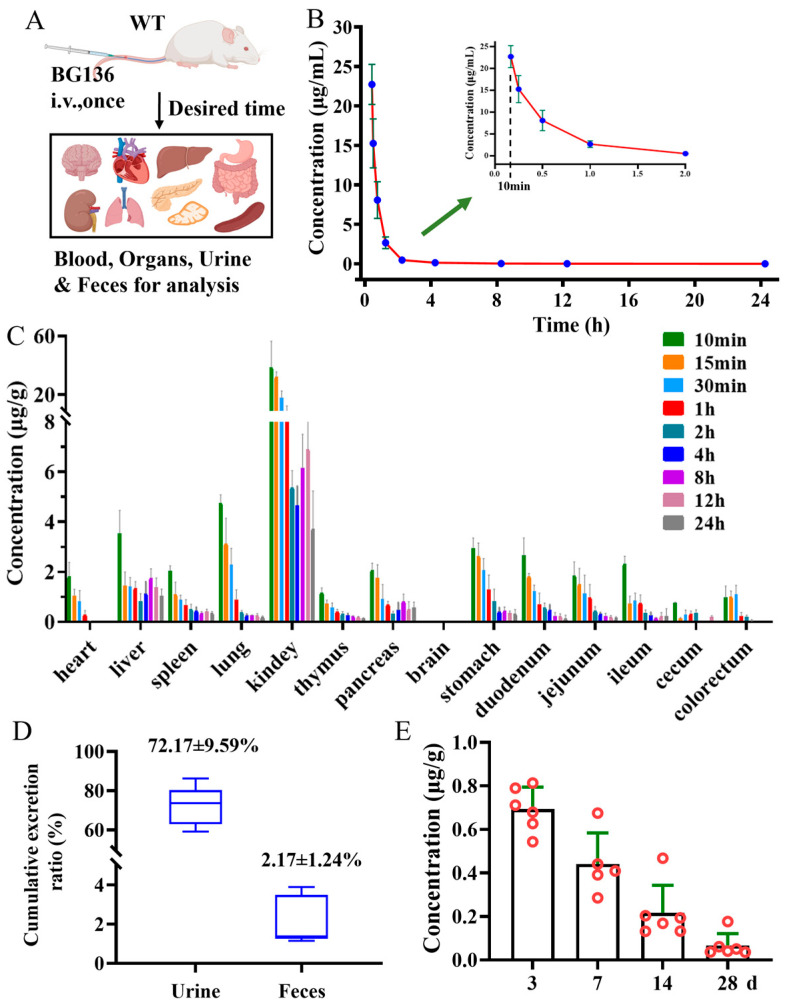
Pharmacokinetic profiles of BG136 in rats after a single injection of BG136. (**A**) Flow chart of rat biospecimen collection, created with BioRender (https://www.BioRender.com); (**B**) mean plasma concentration-time profile; (**C**) tissue distribution of BG136; (**D**) the cumulative excretion rate of BG136 in rat urine and feces; (**E**) long-term disposition of BG136 in the kidney; Data are mean ± SD, *n* = 6.

Following a single intravenous administration, BG136 exhibited a fast elimination pattern in the bloodstream, with a half-life of 1.30 ± 0.42 h (Figure 5B and Table S5). Meanwhile, BG136 was rapidly and non-targetedly trafficked to bodily tissues via systemic circulation (Figure 5C). Subsequently, 72.17 ± 9.59% of the administered dose was excreted in the urine in its parent form via renal excretion within 3 days (Figure 5D). The investigation of a compound’s in vivo persistence is pivotal for evaluating drug efficacy and safety. Previous findings indicated that mice pretreated with β-glucan for 28 days retained anti-tumor activity [31,38]. Yet, few studies have investigated the long-term distribution of polysaccharides in vivo. To figure out whether BG136 accumulates in the body over extended periods, we collected kidneys at 3-, 7-, 14-, and 28-day post-administration for examination. As shown in Figure 5E, BG136 remained detectable at 28 days, confirming its prolonged in vivo disposition and suggesting potential long-term effects.

Since BG136 is excreted through urine, its form in urine can provide direct insight into its in vivo metabolism. As shown in Figure S1, BG136 is mainly eliminated in its unchanged, parent form; however, it remains unclear whether trace amounts of BG136 undergo degradation in vivo. Identifying oligosaccharides resulting from the metabolism of BG136 in biological samples using mass spectrometry poses a significant challenge. This is largely due to the substantial interference from neutral oligosaccharides in biological matrices, coupled with the anticipated low abundance of oligosaccharides derived from BG136 metabolism. To address these issues, we employed an AMAC pre-column derivatization strategy to assess the potential trace degradation of BG136 in vivo. Following a single intravenous administration of 10 mg/kg (2 mg for females and 2.8 mg for males), BG136-derived pentasaccharides and hexasaccharides occurred in urine. Over a 48-h period, their cumulative yields were measured at 2.17 ± 0.99 μg and 5.38 ± 2.17 μg (Figure S5), respectively.

**Figure 6:**
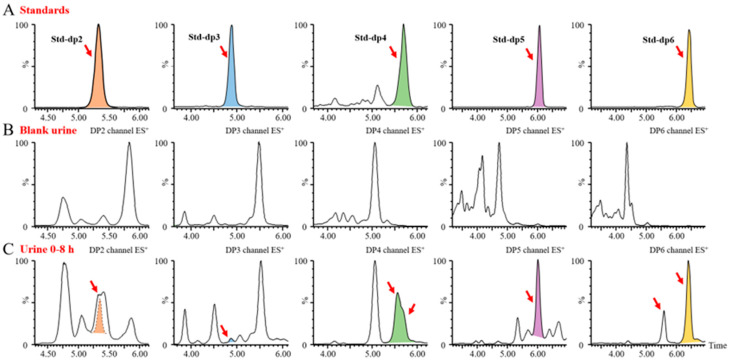
Metabolic oligosaccharide profiles of BG136 in urine. (**A**) Typical chromatograms of oligosaccharides with DP2–6 from BG136 or laminarin; (**B**) oligosaccharide profiles of blank urine; (**C**) oligosaccharide profiles of urine collected at 0–8 h.

Upon increasing the dosage to 100 mg/kg, we identified trace disaccharides, trisaccharides, and tetrasaccharides generated by BG136 degradation, in addition to the previously detected pentasaccharides and hexasaccharides, as illustrated in Figure 6. These findings suggest that trace amounts of BG136 could be degraded to oligosaccharides with DP2–6 in vivo. Compared to high molecular weight β-glucans, BG136 exhibits distinctly different metabolic characteristics. Li et al. reported that PGG, a water-soluble β-glucan with a molecular weight of 150 kDa, was broken down in vivo to a 25 kDa fragment [32]. Similarly, Zheng et al. showed that two triple helical β-glucans from *Lentinus edodes*, with molecular weights of 1160 kDa and 300 kDa, were biodegraded in vivo to a 100-kDa β-glucan active moiety [39]. Indeed, the molecular weights of these generated active fragments were much larger than that of BG136, implying that the metabolic behavior and pattern of action of BG136 may differ significantly from those of high molecular weight β-glucans.

### 2.6. Plasma Pharmacokinetic Profiles in Human Subjects

**Figure 7:**
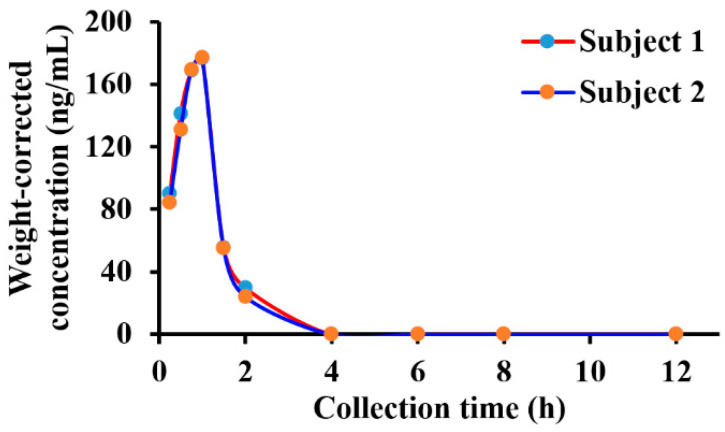
Plasma concentration-time curve in healthy Chinese subjects receiving an intravenous drip of BG136 (2 mg).

In December 2022, BG136 was authorized for clinical trials and is anticipated to be the first immune-antitumor marine polysaccharide drug in China. However, the sensitivity (0.5 μg/mL) of the preclinically established BG136 quantification method proves insufficient for the detection of low-level BG136 in human body fluids at clinically relevant doses. To address this limitation, we developed the present methodology for the sensitive (10 ng/mL) and accurate measurement of BG136 levels in clinical samples. The plasma concentration-time profile of BG136 following a 2 mg intravenous drip in healthy subjects is illustrated in Figure 7. Differences in administration ways between rats and healthy humans led to variations in pharmacokinetic profiles. With intravenous drip, BG136 levels in plasma gradually increased, peaking at 1 h post-administration.

## 3. Materials and Methods

### 3.1. Chemicals and Reagents

BG136 was provided by the Marine Biomedical Research Institute of Qingdao (Qingdao, China). Detailed structural information has been published previously [37]. All the BG136 APIs used in this study were from the same batch (Batch No.: BG136-AT-181016). Agarotriose (purity ≥ 98.0%), serving as an internal standard (IS), was purchased from BZ Oligo Biotech Co., Ltd. (Qingdao, China). endo-1, 3-β-D-glucanase (*Trichoderma* sp.) and glucose oxidase (GOD) were purchased from Megazyme (County Wicklow, Ireland). Sodium cyanoborohydride (purity ≥ 95.0%), HPLC-grade dimethylsulfoxide (DMSO), and LC-MS grade reagents (acetic acid, ammonium acetate, ammonium bicarbonate, methanol, acetonitrile, and isopropanol) were purchased from Sigma-Aldrich (St. Louis, MO, USA). HPLC-grade chloroform and analytical-grade 2-aminoacridone (AMAC, purity ≥ 98.0%) were purchased from Anaqua Chemicals Supply (Shanghai, China) and Macklin (Shanghai, China), respectively. Blank human plasma and urine were obtained from reliable commercial sources.

### 3.2. Animals and Cells

Sprague-Dawley (SD) rats, weighing between 180 to 220 g, were supplied by Vital River Laboratory Animal Technology Co., Ltd. (Beijing, China). The rats were housed in an environment maintained at a temperature of 24 ± 2 °C and a humidity of 65 ± 10%, with free access to water and a commercial diet. Madin-Darby Canine Kidney cells (MDCK), Human Umbilical Vein Endothelial Cells (HUVEC), and RAW264.7 macrophages were purchased from the ATCC institution and cultured in Dulbecco’s modified Eagle’s medium (DMEM) supplemented with 10% fetal bovine serum, 100 U/mL penicillin, and 100 U/mL streptomycin.

### 3.3. Sample Preparation

**Figure 8:**
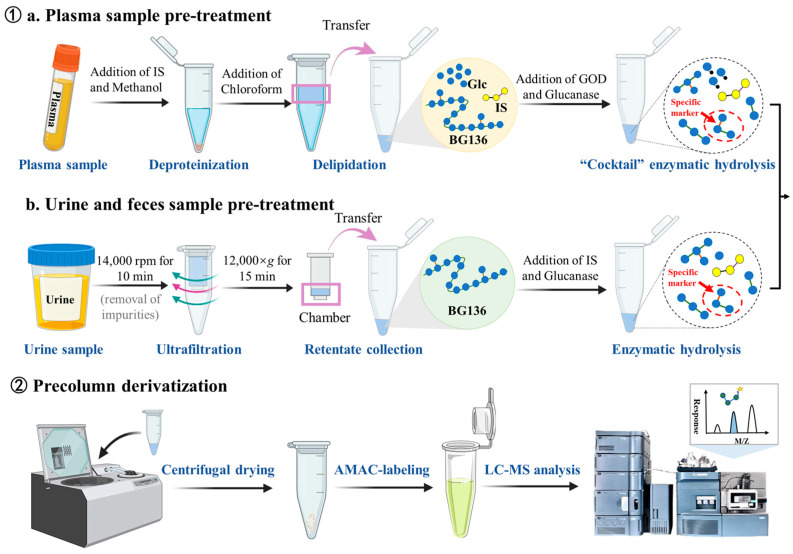
Analytical workflow for quantitative determination of BG136.

The preparation of stock and working standard solutions, calibration standards, and quality control (QC) samples is detailed in the Supplementary Materials. The sample preparation workflow is shown in Figure 8. Briefly, each 50 μL plasma sample was mixed with 50 μL IS working solution—consisting of 25 ng/mL of agarotriose in 500 mM ammonium acetate—and 150 μL methanol for protein precipitation. After vortexing, the mixture was centrifuged at 17,968× *g* (r = 82.0 mm) for 10 min. The resulting supernatant was defatted with 300 μL chloroform through vigorous vortexing (10 min), followed by centrifugation to achieve complete phase separation. The upper aqueous layer was carefully transferred and evaporated to dryness. For enzymatic hydrolysis, 100 μL of enzyme cocktail containing 10 mU endo-1, 3-β-D-glucanase and 100 mU GOD was added to the dried residue at 40 °C for 3 h. The hydrolysates underwent another centrifugal drying process. Derivatization was performed by first adding 20 μL of 50 mM AMAC solution (dissolved in DMSO containing 0.03% acetic acid) and incubating at room temperature in the dark for 5 min, followed by the addition of 20 μL 0.5 M sodium cyanoborohydride solution and incubation at 45 °C for 3 h. The derivatized samples were reconstituted in a diluent (consisting of 80% mobile phase A and 20% mobile phase B, *v*/*v*) and analyzed using LC-MS/MS.

For urine and feces samples, an initial centrifugation was performed at 17,968× *g* for 10 min to separate particulate matter. The supernatant containing BG136 was then subjected to ultrafiltration using a 0.5 mL 3 kDa molecular weight cutoff ultrafiltration column (Millipore, Billerica, MA, USA) through repeated centrifugation at 12,000× *g* for 15 min. The concentrated solution in the insert was collected and hydrolyzed by adding 50 μL of a 150 ng/mL IS solution containing 10 mU of endo-1, 3-β-D-glucanase. Following the reaction, the resulting hydrolysates were dehydrated, derivatized, and analyzed.

### 3.4. Instrumentation and Conditions

The mass spectrometer used in this study was a Waters Xevo TQ-XS outfitted with an ACQUITY UHPLC system (Waters, Milford, MA, USA) and coupled to an electrospray ionization source in positive ionization mode. Samples were chromatographically separated on a Kinetex 2.6 μm Polar LC Column (100 × 2.1 mm) (Phenomenex, Los Angeles, CA, USA), with the column temperature maintained at 45 °C. The mobile phase consisted of a 10 mM ammonium bicarbonate aqueous solution with 0.03% acetic acid (solvent A) and methanol (solvent B) for analyte separation. The gradient elution for plasma samples was conducted as follows: 0.00–6.00 min: 80%A–20%B; 6.01–8.00 min: 0%A–100%B; 8.01–10.00 min: 80%A–20%B. For urine samples, the gradient was adjusted to: 0.00–8.00 min: 82%A–18%B; 8.01–10.00 min: 0%A–100%B; 10.01–12.00 min: 82%A–18%B. The flow rate was set as 0.30 mL/min, with an injection volume of 2 μL. Mass spectrometry acquisition utilized MRM pattern, with transitions of *m*/*z* 689.92 > 374.88 for BG136 and *m*/*z* 681.17 > 374.81 for IS. The optimal cone voltage and collision energy for BG136 were 26 V and 28 eV, respectively, while for IS they were 4 V and 26 eV. Additional MS parameters were configured as follows: capillary voltage at 3.5 kV; desolvation temperature set to 500 °C; desolvation gas flow at 1000 L/h; collision gas flow maintained at 0.15 mL/min; and cone gas flow at 150 L/h. Data processing was carried out using TargetLynx XS software (Version 4.2) provided by Waters (Milford, MA, USA).

### 3.5. Analytical Validation Protocol

The established LC-MS/MS method for quantifying BG136 in various biological matrices including human plasma and urine, rat plasma, urine and feces, was validated in compliance with the 9012 Guidelines for Bioanalytical Method Validation. The following parameters including selectivity, linearity and calibration curve, carryover, accuracy and precision, matrix effect, and stability under various storage conditions were evaluated. Detailed operational procedures and acceptance criteria are outlined in the Supplementary Materials.

### 3.6. Cellular Uptake and Release of BG136

MDCK, HUVEC, and RAW264.7 macrophages were seeded at a density of 1 × 10^6^ cells per well in six-well plates with complete DMEM medium and cultured at 37 °C under a humidified atmosphere of 95% air and 5% CO_2_. For the uptake assay, cells were exposed to various concentrations of BG136 for specified durations. After exposure, the cells were washed several times with PBS buffer to remove free BG136. The cells were then harvested, counted, and lysed using RIPA lysis buffer for quantitative assays. In the release assay, cells were allowed to internalize BG136 for a designated duration, followed by rinsing with PBS buffer. Fresh complete medium was then added, allowing the cells to release BG136 over 12, 24, 36, and 48 h.

### 3.7. Pharmacokinetic Design in Rats

All rats received a single intravenous injection of BG136 at a dosage of 10 mg/kg. Blood samples were drawn into EDTA-K_2_ tubes via the post-orbital venous plexus under brief anesthesia induced by intravenous administration of 25 mg/kg sodium thiopental. Plasma was obtained by centrifuging the blood at 2500× *g* for 15 min. For the tissue distribution experiment, rats were euthanized by intravenous injection of sodium thiopental at a dose of 100 mg/kg. Representative organs were promptly excised and rinsed with saline solution. The tissues were then weighed and homogenized with saline solution at a solid-liquid ratio of 1:3 (*m:v*). After centrifugation, the homogenates were collected. In the excretion assay, urine and feces were collected at specific time intervals: 0–4, 4–8, 8–12, 12–24, 24–36, 36–48, and 48–72 h post-administration. At the end of the experiment, the rats were euthanized. All biological samples were stored at −20 °C until analysis.

### 3.8. Plasma Pharmacokinetic Analysis in Human Subjects

This was a single-dose, open-label study in healthy volunteers at the Drug Clinical Trial Center of the Affiliated Hospital of Qingdao University in China. Written informed consent was obtained from each subject prior to the initiation of the study. Two male subjects were enrolled in the preliminary dosing group (2 mg) and were required to stay in the patient ward one day before dosing to ensure standardized daily life management. During the trial period, the participants refrained from consuming meals containing mushrooms or *Saccharina japonica*. On the following morning, after a blank blood sample was collected, the drug was administered via intravenous drip. Blood samples were collected in EDTA-K_2_-anticoagulant tubes at 0.25, 0.5, 0.75, 1, 1.5, 2, 4, 6, 8, 10, 12, 24, 36, and 48 h post dose. Plasma was separated by centrifugation at 4 °C and stored at −80 °C until analysis.

### 3.9. Statistical Analysis

All data are presented as the mean ± standard deviation (SD). Statistical computations and data visualization were performed using GraphPad Prism 8.0.2.

## 4. Conclusions

In this study, we developed and validated a quantitative methodology based on a structure-specific trisaccharide (Glc-β1, 6-Glc-β1, 3-Glc) to analyze BG136 levels in a wide range of biological samples, including human and rat plasma, urine, feces, tissue homogenates, and cell lysates. This methodology enables the prototype-based BG136 administration and effectively addresses matrix interference, proven to be highly sensitive, accurate, and stable. Moreover, the established quantitative method exhibits broad applicability and great practical value. It allowed for a comprehensive evaluation of cellular internalization and externalization kinetics of BG136 by accurate quantification. Following a single intravenous administration, BG136 exhibited a fast elimination pattern in the bloodstream, leading to rapid distribution to bodily tissues, and remained detectable in vivo for 28 days. Subsequently, 72.17 ± 9.59% of the administered dose was excreted in the urine as a prototype form via renal excretion, and a hint of BG136 was metabolized to oligosaccharides with DP2–6 in vivo. Furthermore, the methodology effectively supported the successful completion of the Phase I clinical trial of BG136. These findings represent the first practical applications of this strategy in cellular, preclinical, and clinical pharmacokinetic studies of polysaccharide-based drugs, providing valuable insights into the efficacy and safety of β-glucan in clinical practice.

## Figures and Tables

**Table 1:** Precision and accuracy of quality control samples for BG136 in plasma, urine, and feces.

Matrix	Sample	Intra-Run (*n* = 6)						Inter-Run (*n* = 18)	
		Run 1		Run 2		Run 3			
		Accuracy (%)	Precision (RSD, %)	Accuracy (%)	Precision (RSD, %)	Accuracy (%)	Precision (RSD, %)	Accuracy (%)	Precision (RSD, %)
Human plasma	LLQC	111.1	4.43	112.3	7.91	96.0	4.50	106.5	9.11
	LQC	97.9	3.03	96.2	4.14	97.4	4.20	97.1	3.66
	MQC	100.1	1.14	93.4	2.44	99.9	1.46	97.8	3.65
	HQC	99.4	1.77	95.4	2.35	103.4	2.08	99.4	3.92
Human urine	LLQC	98.9	6.54	96.4	4.95	104.0	4.27	99.8	5.96
	LQC	100.8	4.15	101.0	4.00	97.5	5.60	99.8	4.64
	MQC	100.1	4.38	98.5	5.17	95.4	9.30	98.3	6.44
	HQC	99.7	7.03	97.7	5.08	103.0	4.93	100.1	5.85
Rat plasma	LLQC	100.2	12.11	107.1	4.01	107.1	7.12	104.8	8.37
	LQC	97.2	7.34	91.6	2.51	94.2	3.93	94.3	5.42
	MQC	105.3	1.31	96.2	4.70	93.5	5.87	98.3	6.63
	HQC	106.9	2.34	99.5	4.38	95.0	4.37	100.5	6.13
Rat urine	LLQC	104.8	4.60	94.8	9.33	106.0	5.63	101.9	8.04
	LQC	102.2	8.63	96.1	3.31	100.0	7.22	99.5	6.96
	MQC	99.6	10.97	93.8	7.52	90.6	5.01	94.7	8.86
	HQC	90.4	6.56	90.1	3.35	95.7	8.29	92.1	6.75
Rat feces	LLQC	100.1	1.02	106.7	5.39	101.1	9.44	102.6	6.60
	LQC	104.1	8.50	105.5	5.44	96.3	9.04	101.9	8.34
	MQC	104.2	10.61	91.7	7.22	92.6	3.80	96.2	9.70
	HQC	105.1	10.28	103.9	9.24	100.6	10.35	103.2	9.55

**Table 2:** Matrix effect of quality control samples for BG136 in human plasma and urine (*n* = 8).

Matrix	Sample	Nominal Conc. (ng/mL)	Matrix Factor (Mean ± SD)		IS-Normalized Matrix Factor	
			BG136	IS	Mean ± SD	RSD (%)
Human plasma	LQC	25	1.08 ± 0.10	1.10 ± 0.03	0.98 ± 0.08	7.85
	HQC	225	1.03 ± 0.19	1.09 ± 0.17	0.95 ± 0.05	5.47
Human urine	LQC	50	17.18 ± 1.37	1.13 ± 0.10	15.34 ± 1.50	9.80
	HQC	750	14.90 ± 0.45	1.04 ± 0.01	14.33 ± 0.45	3.11

**Table 3:** Stability of quality control samples for BG136 in human plasma and urine (*n* = 6).

Matrix	Storage Conditions	Sample	Nominal Conc. (ng/mL)	Recovery (%)	
				Mean ± SD	RSD (%)
Stock solution	Freeze-thaw (−20 °C, 5 cycles)	LQC	25	99.5 ± 5.75	5.78
		HQC	225	94.8 ± 2.81	2.97
Human plasma	4 °C, 20 days	LQC	25	101.2 ± 4.70	4.64
		HQC	225	109.7 ± 1.15	1.05
	Freeze-thaw (−20 °C, 3 cycles)	LQC	25	100.6 ± 2.53	2.52
		HQC	225	102.5 ± 0.90	0.88
	Post-preparative (RT, 14 days)	LQC	25	95.6 ± 7.12	7.45
		HQC	225	102.7 ± 1.86	1.81
Human urine	4 °C, 7 days	LQC	50	108.8 ± 3.22	2.96
		HQC	750	96.8 ± 3.38	3.49
	Freeze-thaw (−20 °C, 5 cycles)	LQC	50	101.7 ± 1.02	1.00
		HQC	750	92.5 ± 4.78	5.16
	Post-preparative (RT, 9 days)	LQC	50	102.7 ± 3.73	3.63
		HQC	750	98.7 ± 7.37	7.47

## Data Availability

Data will be made available upon request.

## Supplementary Materials

The following supporting information can be downloaded at: https://www.mdpi.com/article/10.3390/md23040177/s1, Figure S1: Main existence form of BG136 in urine; Figure S2. Identification of BG136 enzymatic digestion products and structure of purified BG-Tris; Figure S3: Structural characterization of BG-Tris; Figure S4: Influencing factors in the pretreatment process; Figure S5: The cumulative yields of pentasaccharides and hexasaccharides resulting from BG136 degradation in vivo. Table S1: Ion-pair information and optimal MS parameters of standard neutral oligosaccharides; Table S2: Proportion of BG-Tris in aqueous solutions containing different concentrations of BG136; Table S3: Chemical shifts (ppm) of 1D and 2D NMR signals of BG-Tris; Table S4: Standard regression curves of BG136 in various matrices; Table S5: Mean plasma concentration-time profile of BG136 in rats after intravenous injection at 10 mg/kg.

## Abbreviations

The following abbreviations are used in this manuscript:

AMAC	2-Aminoacridone
BG136	β-1, 3/1, 6-glucan
BG-Dis	Disaccharides yielded from BG136
BG-Tris	Trisaccharides yielded from BG136
BG-Tetras	Tetrasaccharides yielded from BG136
DP	Degree of polymerization
GOD	Glucose oxidase
IS	Internal standard
LLOQ	Lower limit of quantitation
MRM	Multiple reaction monitoring
MS	Mass spectrum
QC	Quality control
RSD	Relative standard deviation
TIC	Total ion chromatogram
UHPLC	Ultra-high-performance liquid chromatography
