# Supplementary material for: Exploring the In Vivo Fate of β-1, 3/1, 6-Glucan Using Quantitative Tandem Mass Spectrometry Based on a Structure-Specific Fragment
Source: Mar Drugs. 2025 Apr 20;23(4):177. doi: 10.3390/md23040177 (PMC12028845; doi:10.3390/md23040177)
Supplement: Supplementary file 1 [file marinedrugs-23-00177-s001.zip › marinedrugs-3579338-supplementary.pdf]

Supporting information

Article

Exploring the in vivo fate of β-1, 3/1, 6-glucan using quantitative tandem mass spectrometry based on a structure-specific fragment

Shuying Xu [1], Jiale Hao [1], Chunyan Ye [1], Xintong Li [1], Pengcheng Gao [1], Ni Song [1], Chanjuan Liu [1], Youjing Lv [1], Guangli Yu [1,2,*] and Guoyun Li [1,2,*]

[1] Key Laboratory of Marine Drugs (Ministry of Education), Shandong Key Laboratory of Glycoscience and Glycotherapeutics, School of Medicine and Pharmacy, Ocean University of China, Qingdao 266003, China; 17syxu@alumni.stu.edu.cn (S.X.); hjl9700@163.com (J.H.); ye18716113504@163.com (C.Y.); lixintong20000723@163.com (X.L.); gaopengchengsir@163.com (P.G.); nisong1975@ouc.edu.cn (N.S.); liuchanjuan@ouc.edu.cn (C.L.); lvyoujing1988@163.com (Y.L.)

[2] Laboratory for Marine Drugs and Bioproducts, Qingdao Marine Science and Technology Center, Qingdao266237, China.

* Correspondence: glyu@ouc.edu.cn (G.Y.); liguoyun@ouc.edu.cn (G.L.); Tel.: +86-532-8203-1609 (G.Y.); +86-532-8203-1615 (G.L.)

1. Materials and methods

1.1. Stock and working standard solutions

The stock solutions of BG136 (1.00 mg/mL) and IS (100 μg/mL) were separately prepared by accurately weighing the analytes and dissolving them in distilled water. All stock solutions were stored at -20 ℃. Working IS solutions were obtained by freshly diluting the IS stock solution with 500 mM ammonium acetate solution.

1.2. Preparation of calibration standards and quality control (QC) samples

1.2.1. Plasma Samples

Calibration standards and QC samples were prepared by diluting the BG136 stock solution with drug-free human plasma. The pre-set concentration levels of calibration standards were 250, 200, 150, 100, 50 and 10 ng/mL. High concentration QC (HQC; 225 ng/mL), medium concentration QC (MQC; 125 ng/mL), low concentration QC (LQC; 25 ng/mL) as well as lower limit of quantitation (LLOQ; 10 ng/mL) samples were prepared by diluting the BG136 stock solution with blank plasma.

1.2.2. Urine Samples

As with the procedure above, calibration standards and QC samples were prepared by diluting the BG136 stock solution with blank human urine. The calibration samples were obtained by gradient dilution with blank urine, and the final concentrations were 1000, 800, 600, 400, 200, 100, 60 and 20 ng/mL, respectively. In the same way, HQC (750 ng/mL), MQC (500 ng/mL), LQC (50 ng/mL) as well as LLOQ (20 ng/mL) samples were prepared by diluting the BG136 stock solution with blank urine.

1.2.3. Feces Samples

The water-free rat fecal sample was accurately weighed, and normal saline was added (solid-liquid ratio of 1: 50). After a 10-minute swirl, the suspension was centrifuged. The obtained supernatant was defined as a blank fecal solution. Calibration standards and QC samples were prepared by diluting the BG136 stock solution with blank fecal solution. The given concentration levels of calibration standards were 1000, 800, 600, 400, 200, 100 and 50 ng/mL. HQC, MQC, LQC and LLOQ fecal samples were 750 ng/mL (37.5 μg/g), 500 ng/mL (25.0 μg/g), 140 ng/mL (7.0 μg/g), and 50 ng/mL (2.5 μg/g), respectively.

1.3. Method Validation

1.3.1. Selectivity

The selectivity of the method was assessed by analyzing blank matrices from at least six different individuals and identical matrices spiked with BG136 at the LLOQ level. The assay is considered acceptable only when the area of the interference peak at the same retention time of analytes is less than 20% respond of the LLOQ for BG136 and 5% respond of the IS.

1.3.2. Calibration Curve and Linearity

Linearity was evaluated by least-squares analysis of the calibration curve based on peak area ratio of BG136 to IS (Y) versus the concentration of BG136 (X, ng/mL) weighted using 1/X. Linearity is recognized when the correlation coefficient (r2) is > 0.99 and the calculated concentration is within ± 15% of the theoretical value (± 20% at the LLOQ).

1.3.3 Carryover

The residual effect was evaluated by injecting a blank sample following the analysis of a high-concentration plasma or urine sample of BG136. The acceptance criteria stipulate that BG136 residual peak area should not exceed 20% of the LLOQ, while the IS residual peak area should not exceed 5% of the IS response.

1.3.4. Precision and Accuracy

Intra-run (an analytical batch) and inter-run (three analytical batches) accuracy and precision were assessed by analyzing six replicates of QC samples at the LLOQ, LQC, MQC and HQC levels. Accuracy (%) was calculated as: measured concentration/nominal concentration × 100%, and precision was defined as the relative standard deviation (RSD, %) at each concentration level. The mean value of accuracy should be within 85-115% of the nominal value and the RSD of precision should not exceed 15% at the LQC, MQC and HQC levels, and 20% at the LLOQ level.

1.3.5. Matrix effect

Matrix effect was evaluated by comparing the peak responses of analytes dissolved in the blank biological matrix with those dissolved in distilled water at the same concentration. Matrix effect should be measured at LQC and HQC concentrations. The criteria for acceptability of the coefficient of variation of the IS normalized matrix factors calculated from six batches of matrix should be ≤ 15%.

1.3.6. Stability

The stabilities of analytes in human plasma and urine were tested by analyzing six replicates at low and high concentrations after being exposed to different conditions. Freeze and thaw stability was manipulated by refreezing at -20 ℃ for 24 h and then thawing at room temperature for at least three cycles. Short-term stability at 4 ℃ was tested by keeping samples in a 4 ℃ refrigerator for a minimum of 7 days. Post-preparative stability was evaluated by analyzing processed samples stored at ambient temperature for at least 9 days. The mean of the observed values should be within ± 15% of the nominal value.

2. Results and discussion

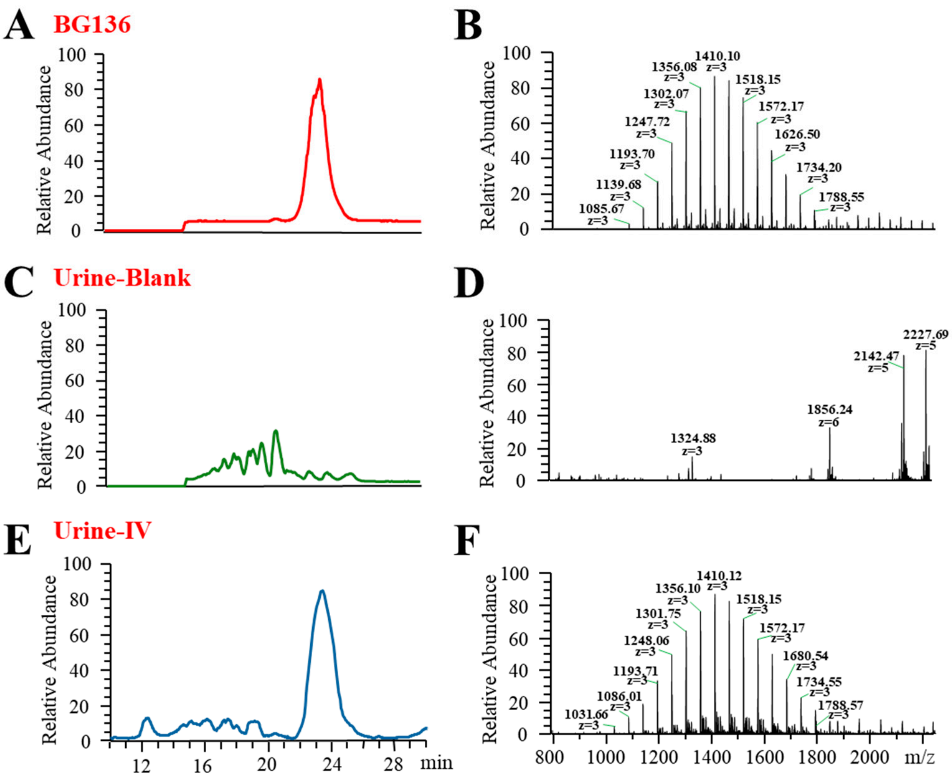

Figure S1. Main existence form of BG136 in urine. (A-B) TIC chromatogram and mass profile of BG136 standard; (C-D) TIC chromatogram and mass profile of blank urine; (E-F) TIC chromatogram and mass profile of urine collected after treatment.

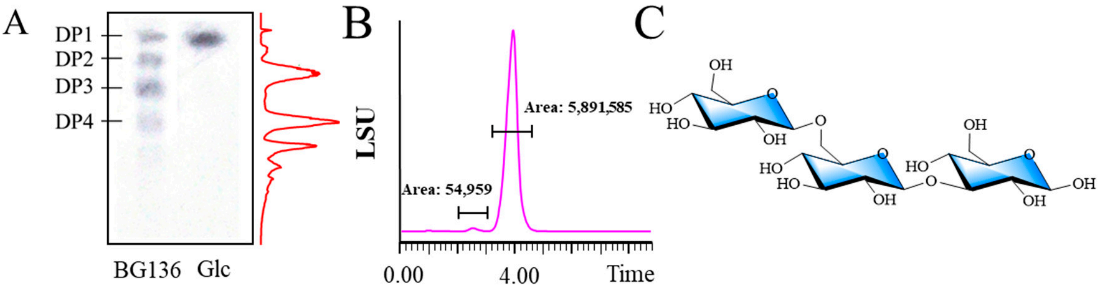

Figure S2. Identification of BG136 enzymatic digestion products and structure of purified BG-Tris. (A) TLC profile of the enzymatic product of BG136; (B) purity analysis of BG-Tris obtained after isolation and purification of the enzymatic product; (C) structure of BG-Tris.

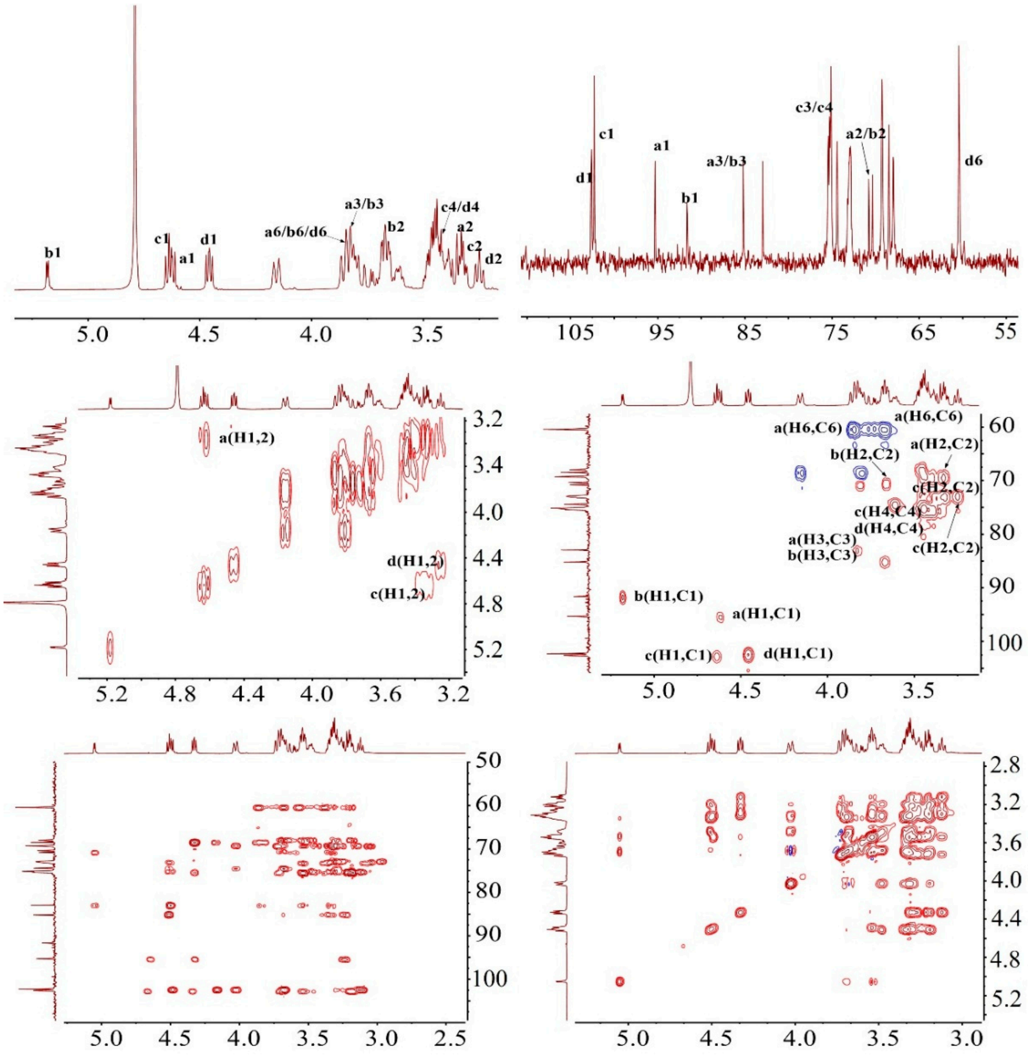

Figure S3. Structural characterization of BG-Tris. (A-B) ^{1}H NMR and ^{13}C NMR; (C-F) COSY, HSQC, HMBC and TOCSY spectra, respectively.

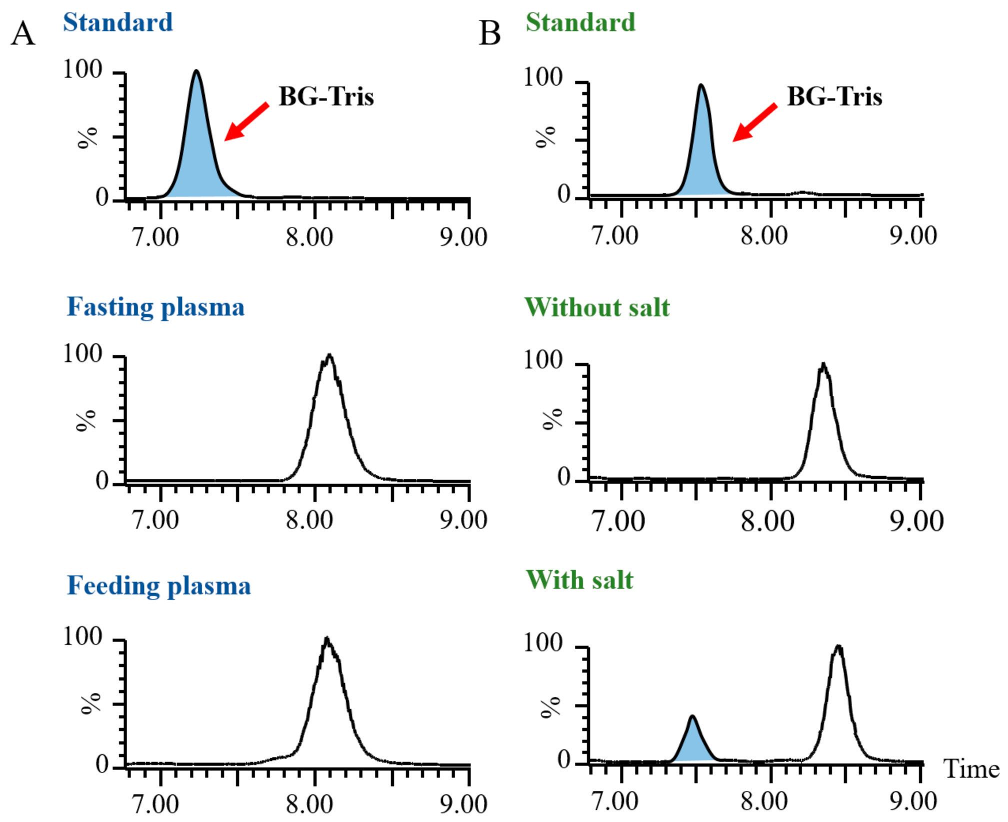

Figure S4. Influencing factors in the pretreatment process. (A) Impact of diet on detection of BG136 in human plasma; (B) necessity of adding salt solution in the process of protein removing.

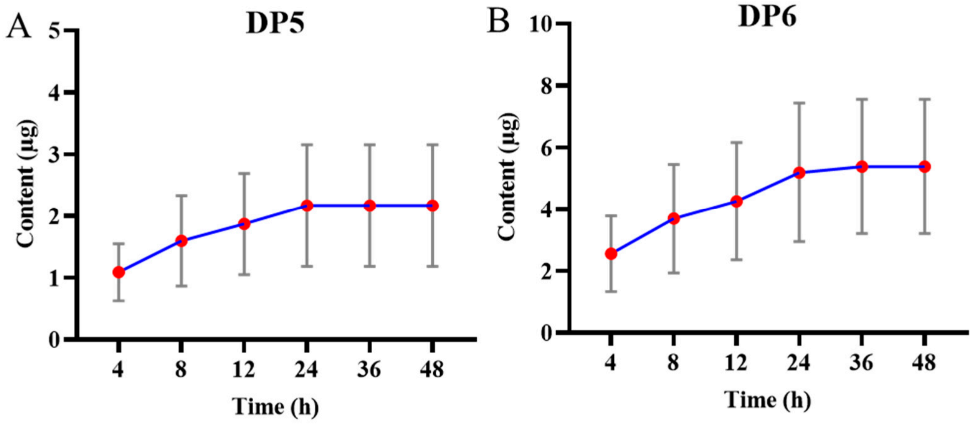

Figure S5. The cumulative yields of pentasaccharides (A) and hexasaccharides (B) resulting from BG136 degradation in vivo; Data are mean ± SD, n=6.

Table S1. Ion-pair information and optimal MS parameters of standard neutral oligosaccharides.

Standards	Precursor (m/z)	Daughter (m/z)	Cone voltage (V)	Collision energy (eV)
Disaccharide	536.87	374.88	28	16
Trisaccharide	689.92	374.88	26	28
Tetrasccharide	861.03	374.81	10	30
Pentasaccharide	1023.34	374.75	64	36
Hexasaccharide	1185.40	374.79	68	44
Agarotriose (IS)	681.17	374.81	4	26

Table S2. Proportion of BG-Tris in aqueous solutions containing different concentrations of BG136.

Sample	Nominal concentration of BG136 (ng/mL)	Measured concentration of BG-Tris (ng/mL)	% Proportion	Mean±SD
BG136-S0	0	0.261	-	
BG136-S1	10	3.424	34.2	
BG136-S2	50	17.674	35.3	
BG136-S3	100	34.557	34.6	35.48±0.97%
BG136-S4	150	55.222	36.8	
BG136-S5	200	71.937	36.0	
BG136-S6	250	89.966	36.0	

Table S3. Chemical shifts (ppm) of 1D and 2D NMR signals of BG-Tris.

Residue	H1/C1	H2/C2	H3/C3	H4/C4	H5/C5	HC/C6
→3)-β-Glc (β)	4.61~4.62/95.34	3.33/70.89	3.82/85.22	-	-	3.84,3.69~3.65/62.07
→3)-β-Glc (α)	5.18/91.57	3.66/70.81	3.82/85.22	-	-	3.84,3.69~3.65/62.07
→6)-β-Glc(1→	4.63/102.56	3.32/74.70	-	3.42/76.02	-/76.75	3.84,3.69~3.6/70.10
β-Glc(1→	4.45/102.30	3.25/74.53	-	3.42/77.05	-	3.84,3.69~3.65/62.07

Table S4. Standard regression curves of BG136 in various matrices.

Matrix	Linear range	Calibration curve	Correlation coefficient (r^2)
Human plasma	10-250 ng/mL	Y=0.0019X+0.00241	0.9991
	250-80000 ng/mL	Y=0.0031X+0.14446	0.9981
Human urine	20-1000 ng/mL	Y=0.0033X+0.0098	0.9979
Rat plasma	10-1000 ng/mL	Y=0.0087X+0.3204	0.9986
	1000-80000 ng/mL	Y=0.0093X+1.4480	0.9978
Rat urine	20-1000 ng/mL	Y=0.0016X+0.1017	0.9952
	1000-80000 ng/mL	Y=0.0013X-0.0940	0.9986
Rat feces	2500-50000 ng/g	Y=0.0004X-0.0147	0.9931
Heart	60-3000 ng/g	Y=0.0021X+0.2893	0.9943
Liver	60-3000 ng/g	Y=0.0017X+0.0180	0.9968
Spleen	60-3000 ng/g	Y=0.0021X+0.0272	0.9975
Lung	60-3000 ng/g	Y=0.0017X+0.0148	0.9960
Kidney	60-3000 ng/g	Y=0.0015X+0.0212	0.9985
Thymus	60-3000 ng/g	Y=0.0017X-0.0135	0.9971
Pancreas	60-3000 ng/g	Y=0.0014X+0.0484	0.9969
Brain	60-3000 ng/g	Y=0.0012X+0.1365	0.9954
Stomach	60-3000 ng/g	Y=0.0017X+0.0431	0.9968
Duodenum	60-3000 ng/g	Y=0.0013X+0.0425	0.9942
Jejunum	60-3000 ng/g	Y=0.0017X-0.0064	0.9975
Ileum	60-3000 ng/g	Y=0.0017X+0.0170	0.9941
Cecum	60-3000 ng/g	Y=0.0012X+0.0386	0.9933
Colorectum	60-3000 ng/g	Y=0.0011X+0.0377	0.9937
Cell lysate	10-1000 ng/mL	Y=0.0020X-0.0105	0.9973

Table S5. Mean plasma concentration-time profile of BG136 in rats after intravenous injection at 10 mg/kg (n=6).

The parameters	Mean ± SD
$T_{1/2}$ (h)	1.30 ± 0.42
T_{max} (h)	0.17 ± 0.00
C_{max} (μg/mL)	22.72 ± 2.54
AUC_{0-t} (h*μg/mL)	16.19 ± 2.46
$AUC_{0-\infty}$ (h*μg/mL)	16.24 ± 2.46
Vss (mL/kg)	361.01 ± 59.55
Cl (mL/h/kg)	628.52 ± 95.48
MRT_{0-t} (h)	0.54 ± 0.07
$MRT_{0-\infty}$ (h)	0.58 ± 0.06
